# Reflective practice and health sciences librarians: engagement, benefits, and barriers

**DOI:** 10.5195/jmla.2020.777

**Published:** 2020-01-01

**Authors:** Jolene M. Miller

**Affiliations:** Director, Mulford Health Science Library, and Assistant Professor, Library Administration, University of Toledo, Toledo, OH, jolene.miller@utoledo.edu

## Abstract

**Objective:**

Reflective practice is common in nursing and other professions. In the published literature, there is very little about librarians’ use of reflective practice and no studies of health librarians’ use of reflective practice. This study examined the use of reflective practice among health sciences librarians, perceived benefits, and perceived barriers to use.

**Methods:**

This cross-sectional study replicated the 2014 study by Greenall and Sen, using a version of their questionnaire. The research population in this study was health sciences librarians who were members of the MEDLIB-L email discussion list, Medical Library Association (MLA) chapter email discussion lists, and/or MLA section email discussion lists.

**Results:**

There were 106 librarians who completed the questionnaire, ranging from those new to the profession through midcareer to longtime librarians. While a high percentage of respondents considered themselves to be reflective practitioners (77%), a larger percentage (87%) reported that they consciously spent time reflecting. Respondents selected a wide variety of benefits of reflective practice, while barriers tended to center on lack of time, knowledge, skills, or experience.

**Conclusion:**

The diversity of benefits that respondents selected suggests that reflective practice can play an important positive role in librarians’ professional development. Reported barriers to reflective practice suggest that there is a need for educational opportunities to develop skills.

## INTRODUCTION

For most people, reflection is a daily process. We think back on a situation, actions taken or not taken, words said or not said, reactions, and outcomes. This may be triggered by external events (such as a patron encounter) or internal events (feeling uneasy after a meeting). Reflection has been defined as “a process of thinking, feeling, imagining, and learning by considering what has happened in the past, what is currently happening, and what could possibly happen in the future” [1]. While informal reflection is commonplace, it does not necessarily result in changes in thinking or behavior that translate into improved performance-related outcomes. This has led practitioners and researchers in a variety of fields to explore reflection with the explicit purpose of improving performance: reflective practice.

Reflection as a part of professional practice was popularized by Donald Schön’s 1983 book, *The Reflective Practitioner: How Professionals Think in Action* [2]. While his focus was on reflection-in-action (how professionals reflect while they are acting), he also described reflection-on-action, when reflection takes place after the fact. Published works on reflective practice usually refer to reflection-on-action. Much of the literature of reflective practice originated in professions that rely heavily on interpersonal interactions in complex settings, such as nursing, education, and social work. The purpose of the current study is to extend knowledge of the use of reflective practice in librarianship, another profession that is rich in interpersonal interactions in complex settings.

Reflective practice takes a variety of formats. Sometimes, it is an individual pursuit: pondering or writing in a journal. Sometimes it is practiced with others: talking with a trusted mentor or participating in a reflective practice group. It can also be a combination of both, with conversation following individual reflection. It may be freeform or follow a model. It includes consideration of objective facts of the situation (who was involved, what happened) with subjective information (how one felt, how one thinks others felt). Reflection may be supplemented with additional documentation, such as feedback from others. Critical reflective practice adds a consideration: examination of one’s own assumptions about professional practice as well as assumptions of the profession and the broader culture [3]. Despite the variety of techniques, the goal is the same: improving practice by identifying strengths and areas for improvement, changing processes, improving interpersonal and other skills, and increasing self-awareness and self-understanding.

### Reflective practice in librarianship

Professional library and information organizations support reflective practice among their members, but how they do that varies. Reflective practice is part of statements on professional development [4–6] and professional competencies [7]. It has been integrated into continuing education (CE) programs [8], professional registration [9], and credentialing [10]. Reflective practice is also a component of evidence-based library and information practice: “It is up to individual practitioners to be actively reflective in their practice so that they recognize problems and potential solutions sooner and can trace progress in their own decision making within the context of their organization” [11].

The Association of College & Research Libraries (ACRL) promotes reflective practice in its standards for information literacy librarians [7, 12]. In 2018, its Information Literacy Immersion Program included critical reflection as one of its four cornerstones, with two specific objectives: first, “to engage in critical reflective practice in order to explore information literacy, teaching practices, leadership, and the educational role of librarians in higher education”; and second,” to examine issues of power, privilege, and empowerment in teaching, learning, and higher education through the lenses of critical theories and pedagogies in order to develop a personal commitment to inclusive and accessible instructional practice”[13]. The transition from a focus on specific models of teaching and learning to one of critical reflective practice provides a “more flexible foundation for making decisions about their practice, at whatever level, such as the classroom, an instruction program, or a lesson plan” [14].

In health sciences librarianship, the Medical Library Association (MLA) has promoted reflective practice as a part of a culture of research: “Ours will be a profession of reflective practitioners (those who thoughtfully consider their own experiences and apply this knowledge to practice), where evidence is identified, applied, and assessed in a continuous loop of quality improvement with research as the critical underlying construct” [15]. In addition, MLA requires annual reflective assessments for Provisional Members of the Academy of Health Information Professionals (AHIP). The new professional compares current and future positions to MLA’s *Competencies for Lifelong Learning and Professional Success*, first individually, then in conversation with an assigned mentor [10]. An updated version of professional competencies, enhanced with basic and expert performance indicators, was released in 2017 [16], together with an online competency self-assessment [17]. This free self-assessment provides structure to guide reflection and has been widely promoted for all medical and health sciences librarians, regardless of the length of time they have been in the profession.

The published literature on reflective practice in librarianship is diverse. A 2007 systematic review analyzed articles on reflection published in the library literature [18], noting the trend of earlier articles being more descriptive with more recent articles including analytical reflection of reflective practice. Newer articles have explored the use of reflective practice in library and information science education, from either the perspective of developing students’ reflective practice skills [19, 20] or using reflective practice to improve alignment of objectives, expectations, and needs in library and information science coursework [21]. Other articles looked at the use of reflective learning with students in an information literacy instruction setting [22, 23], though as Reale points out, “librarians, if they want to use reflection with their students, should be reflective practitioners themselves” [23].

Published work on librarians’ use of reflective practice to improve teaching skills and teaching identity has described using a reflective teaching journal alone [24] or combined with additional data sources [25]. Andretta reported on the integration of reflective practice into a course developed to help National Health Service librarians develop skills in information literacy education [8]. Macdonald describes the role that critical reflective practice played in her “internal transition from accidental teaching librarian to information literacy educator” [26]. She “questioned the self-perceptions and taken-for-granted attitudes that can become barriers to professional growth and development.”

Interest in reflective practice in librarianship continues. At the 2019 ACRL conference, there were sessions on the use of reflective practice to enhance statements of teaching philosopy, to clarify professional identify, to help faculty improve student assignments, to improve assessment of student assignments, and to improve librarian teaching. At MLA ’19, one of the immersion sessions focused on reflective practice.

Only one article has explored the use of reflective practice by library and information professionals [27]. In 2014, Greenall and Sen surveyed librarians and information professionals in Great Britain to determine the extent to which they use reflective practice in their work. Greenall and Sen asked about two forms of reflection: reflective practice (“thinking alone, sharing in group discussion with colleagues, or talking to one other person”) and reflective writing (“recording reflections, which can be paper or electronic”). For each type of reflection, respondents were asked if they used it, if they had received training in it, what they perceived the benefits of it were (Table 1), and what they perceived the barriers to it were (Table 2). The lists of benefits and barriers were derived from reviews of published literature.

**Table 1:** Perceived benefits of reflective practice and writing

Benefit
Continuing professional development
Learning from significant incidents
Learning from training or educational opportunities
Identification of gaps in skills and knowledge
Identification of personal strengths and weaknesses
Identification of goals
Increasing knowledge
Increasing understanding
Linking theory and practice
Improving planning of future actions
Improving professional judgments
Improving critical thinking
Solving dilemmas
Achieving perspective
Achieving clarity
Understanding and expressing emotions
Managing stress
Understanding the perspective of others
Improving working relationships
Improving professional practice
Identify the need to change
Catalyst for change
Personal empowerment
Emancipation
Self-development
Appreciation of achievements
Sharing experiences with others
Demonstrating professional practice to others
Other

**Table 2:** Perceived barriers to reflective practice and writing

Barrier
Lack of time
Lack of knowledge
Lack of guidance
Lack of training
Lack of skill
Lack of experience
Lack of motivation
Concerns about confidentiality
No perceived benefits of reflection
Negative impact on self-esteem
Unwillingness to focus on emotions
Unwillingness to admit mistakes
Fear of repercussions
Not supported by organizational culture
Other

The study did not ask about critical reflection as a separate category, though some of the benefits (such as “Personal empowerment” and “Emancipation”) suggested critical reflective practice. The survey received 424 responses, with 92% of the respondents indicating that they identified as reflective practitioners. More than half (52%) reported engaging in reflective writing.

The current study extended this line of research by modifying the Greenall and Sen questionnaire to gather information on how health sciences librarians, primarily in the United States, use reflective practice and/or reflective writing, how they learned to practice it, what the perceived benefits of its use were, and what the perceived barriers to its use were. Specific research questions of this study include:

1. Do medical/health sciences librarians engage in reflective practice and/or reflective writing?
2. Are there differences in reported engagement in reflective practice and/or writing in terms of work environment?
3. Are there differences in reported engagement in reflective practice and/or writing among librarians who have received formal training in reflective practice versus those who have not had training?
4. Are there differences between those who engage in reflective practice and/or writing versus those who do not, in terms of perceived benefits or perceived barriers?
5. Are there differences between respondents who have received formal training in reflective practice versus those who have not had training, in terms of perceived benefits or perceived barriers?

## METHODS

The purpose of this cross-sectional study was to describe the use of reflective practice among health sciences librarians. It replicated the 2014 Greenall and Sen study of British library and information professionals [27]. The University of Toledo Social, Behavioral, and Educational Institutional Review Board determined the study to be exempt from institutional review board oversight on March 20, 2017 (SBE IRB #: 201926).

### Instrument

The 2014 Greenall and Sen questionnaire was slightly modified for this study, including changes to demographic questions so that the response options were more relevant to health sciences librarians (supplemental Appendix A). In addition, questions were added to gather information about how respondents developed skills in reflective practice and reflective writing. The definitions of reflective practice (“thinking alone, sharing in group discussion with colleagues, or talking to one other person”) and reflective writing (“recording reflections, which can be paper or electronic”) were maintained in the current study. The questionnaire asked respondents to identify benefits of and barriers to reflective practice and writing. The lists of benefits and barriers were used without modification from Greenall and Sen (Tables 1 and 2). Three open-ended questions were included to gather additional information about reflective practice and reflective writing.

### Population

The research population for this study was health sciences librarians, primarily those working in the United States. To maximize participation in the study, email invitations were sent to thirty-five US-centered, health sciences librarian–related email lists.

### Recruitment

In April 2017, study participants were recruited via emails to email lists for medical and health sciences librarians: MEDLIB-L, MLA chapter email lists, and MLA section email lists. Emails sent to these lists described the research and invited health sciences librarians to complete the online questionnaire.

### Data analysis

Descriptive and chi-square statistics were performed using IBM SPSS Statistics, version 24, 2016. Three open-ended questions (two expansion questions and one general question) were manually reviewed for themes.

## RESULTS

There were 106 responses to the questionnaire. It was impossible to calculate the response rate because of the inability to deduplicate email addresses from 35 email lists with access to few subscriber lists. Comparisons for the research questions in common between this study and the 2014 Greenall and Sen study are described in the “Discussion” section. The “Results” section uses the word “expected” to refer to the predicted values as calculated in the chi-square analyses.

### Do medical/health sciences librarians engage in reflective practice and/or reflective writing?

Most respondents, 94 (88.7%), engaged in reflective practice and/or reflective writing. Eighty-two (77.4%) indicated that they considered themselves to be reflective practitioners all or some of the time. Of the 24 respondents who did not identify as reflective practitioners, 10 (nearly half) reported spending time reflecting on their professional practice. Respondents were considered to be engaged in reflective practice if they reported engaging in conscious reflection at least sometimes, regardless of identification as a reflective practitioner. In total, 92 (86.8%) respondents engaged in conscious reflection at least sometimes. In terms of reflective writing, 52 (49.1%) reported engaging in reflective writing at least sometimes. Of those participants who used reflective writing, 19 (36.5%) reflected on paper only, 11 (21.2%) reflected electronically only, and 22 (42.3%) reported using both. More than half of the respondents, 32 (61.5%), did not share their writings, while 17 (32.7%) shared them with selected people, such as a mentor.

### Are there differences in reported engagement in reflective practice and/or writing in terms of work environment?

Respondents were asked to identify their current work environments. The largest group of respondents, 42 (39.6%), worked in academic health centers; 31 (29.2%) worked in hospitals or medical centers, with general college or university librarians constituting the third-largest group, 13 (12.3%). For analysis purposes, environments with less than 10 responses (medical or health associations or societies, government, self-employed or consultant, research centers, currently not employed, and no answer) were clustered into a fourth group of 11 (10.4%). The work environments that were not selected by any respondents were nursing or allied health schools and schools of library and information studies.

Librarians in hospitals or medical centers reported slightly lower than expected participation in reflective practice (25 actual versus 27.2 predicted). Librarians in the other 3 work environments reported slightly higher participation in reflective practice than expected (academic health center: 38 actual versus 36.8 predicted; college or university: 12 actual versus 11.4 predicted; other environments: 10 actual versus 9.6 predicted). Differences were not statistically significant. There was a similar pattern of non–statistically significant differences among librarians participating in reflective writing, with hospital or medical center librarians reporting slightly lower than expected participation (14 actual versus 16 predicted) and the other 3 groups reporting slightly higher than expected participation.

### Are there differences in reported engagement in reflective practice and/or writing among librarians who have received formal training in reflective practice versus those who have not had training?

While 92 (86.8%) respondents reported reflecting on professional practice at least some of the time, only 7 (6.6%) respondents reported having had formal training in professional or personal reflective practice. The most commonly selected formal training program was a CE program offered by a professional association (selected by 3 of the 7 respondents). Library school and personal development programs came in second (each selected by 2 respondents). All 7 respondents indicated that they also learned about reflective practice through self-directed, independent learning. In addition to those reporting formal training, 8 (7.5%) respondents indicated that they learned about reflective practice on their own. In total, 15 (14.2%) of the 106 respondents learned about reflective practice through independent learning.

In terms of reflective writing, only 8 (7.5%) of the respondents reported having had formal training. The most commonly identified setting for training was working with a mentor (8 respondents), followed by CE in the workplace (3 respondents). Library school, non–master’s of library and information science (MLIS) academic programs (as mentioned in respondent comments), and personal development programs were each selected by 2 respondents. These 8 respondents each indicated that they also used self-directed independent learning to develop their skills in reflective writing. In addition to those reporting formal training, 1 participant identified learning through self-directed, independent learning for a total of 9 (8.5%).

Training in reflective practice or writing appeared to be associated with a slightly increased likelihood of reflecting on professional performance using reflective practice or reflective writing (27 actual versus 25.1 predicted), but this association was not found to be statistically significant. Some respondents used the open-ended questions to express a desire to learn more. Some indicated that they were new to reflective practice: “Reflective practice in librarianship is a new concept for me. I would like to have some training in this” and “Would love to learn more. Maybe a reflective practice lite version?” Others had experience and were curious to learn more about specific aspects of the process: “I do it informally and without labelling it as such; I would appreciate some training into what it is and how to work it into daily practice” and “I engage in something more like ‘ruminative practice’ where I dwell on past mistakes. I would be interested to learn if true reflective practice also includes past successes to determine how they can affect future work.”

### Are there differences between those who engage in reflective practice and/or writing versus those who do not, in terms of perceived benefits or perceived barriers?

Respondents were asked to identify benefits of engaging in reflective practice and writing from a list of 28 benefits (Table 1); they could select all that applied. Overall, the top 5 identified benefits of reflective practice were “Learning from significant incidents” (n=90, 84.9%); “Identification of personal strengths and weaknesses” (n=86, 81.1%); “Identification of gaps in skills and knowledge” (n=79, 74.5%); “Achieving perspective” (n=78, 73.6%); and “Improving planning of future actions” (n=75, 70.8%). The top 5 identified benefits of reflective writing were “Identification of personal strengths and weaknesses” (n=70, 66.0%); “Learning from significant incidents” (n=69, 65.1%); “Achieving perspective” (n=65, 61.3%); “Increasing understanding” (n=63, 59.4%); and “Achieving clarity” (n=60, 56.6%).

There was consistency across types of work environments (supplemental Appendix B). When the rankings were broken down by library type, two benefits appeared in the top ranked lists (top five ranked benefits, with ties resulting in lists of more than five) of all four library types for combined reflective practice or writing: “Identification of personal strengths and weaknesses” and “Achieving perspective.” “Identification of gaps in skills and knowledge” and “Learning from significant incidents” appeared on three of the four lists.

Respondents’ comments highlighted the multiple levels that reflective practice or writing benefits: the librarian, the patron, and the institution. For example, “I personally find it extremely valuable and I find it to yield important insights, both into my own practices and so forth and into how users I support do their jobs and what their needs are” and “There is always room for growth, and reflective practice gives you documentation for that journey for your benefit, and adds to the institutional knowledge/history.”

Respondents were also asked to select all the barriers that prevented them from engaging in reflective practice and writing from a list of 14 specific barriers (Table 2). The top 5 barriers to reflective practice were “Lack of time” (n=73, 68.9%); “Lack of training” (n=44, 41.5%); “Lack of guidance” (n=39, 36.8%); “Lack of knowledge” (n=38, 35.8%); and “Not supported by organizational culture” (n=37, 34.9%). For reflective writing, the most commonly selected barriers were “Lack of training” (n=39, 36.8%); “Lack of time” (n=34, 32.1%)*; “Lack of knowledge” (n=33, 31.1%); “Lack of guidance” (n=30, 28.3%); and “Lack of motivation” (n=27, 25.5%). There was also consistency in identified barriers across work environments (supplemental Appendix B). Four barriers appeared in the top ranked lists (top 5 ranked barriers, with ties resulting in lists of more than 5) of all library type groups for combined reflective practice or writing: “Lack of time,” “Lack of training,” “Lack of knowledge,” and “Lack of guidance.”

Engagement in reflective practice and/or writing appeared to have a positive impact on a respondents’ overall perception of reflection, as represented by identification of benefits. Respondents who engaged in reflective practice selected each of the 28 benefits more often than expected compared to those who did not reflect. There were statistically significant differences between those who engaged in reflective practice and those who did not for 6 benefits: “Identification of gaps in skills and knowledge” (74 actual versus 70.3 predicted; χ^2^(1)=6.245, *p*=0.012); “Identification of personal strengths and weaknesses” (79 actual versus 76.4 predicted; χ^2^(1)=4.015, *p*=0.045); “Increasing knowledge” (49 actual versus 45.1 predicted; χ^2^(1)=4.927, *p*=0.026); “Improving planning of future actions” (71 actual versus 66.8 predicted; χ^2^(1)=7.200, *p*=0.007); “Achieving clarity” (66 actual versus. 62.5 predicted; χ^2^(1)=4.652, *p*=0.031); and “Identify the need to change” (61 actual versus 57.3 predicted; χ^2^(1)=4.839, *p*=0.028).

Likewise, respondents who engaged in reflective writing tended to select benefits more often than expected than those who did not for 26 of the 28 benefits. Those who were *not* engaged in reflective writing selected “Managing stress” (31 actual versus 30.6 predicted) and “Demonstrating professional practice to others” (10 actual versus 8.7 predicted) more often than expected; neither of these were statistically significant. Of the 26 benefits selected more often than expected by librarians engaged in reflective writing, 6 benefits were statistically significant: “Continuing professional development” (21 actual versus 15.7 predicted; χ^2^(1)=5.035, *p*=0.025); “Learning from significant incidents” (43 actual versus 34.3 predicted; χ^2^(1)=12.624, *p*=0.000); “Increasing understanding” (37 actual versus 31.4 predicted; χ^2^(1)=4.955, *p*=0.026); “Improving planning of future actions” (32 actual versus 26.0 predicted; χ^2^(1)=5.436, *p*=0.020); “Improving professional judgments” (30 actual versus 23.1 predicted; χ^2^(1)=7.374, *p*=0.007); and “Improving working relationships” (22 actual versus 16.2 predicted; χ^2^(1)=5.946, *p*=0.015).

With respect to barriers, respondents who had engaged in reflective practice selected fewer barriers more often than expected, only 4 of the 14 barriers: “Lack of motivation,” “No perceived benefits of reflection,” “Negative impact on self-esteem,” and “Fear of repercussions.” For these barriers, the differences between actual numbers versus predicted numbers were 0.8 or less, and none were statistically significant. Only 2 barriers had statistically significant differences between respondents who had engaged in reflective practice and those who had not. Those who were *not* engaged in reflective practice were more likely than expected to identify “Lack of knowledge” (9 actual versus 5.3 predicted; χ^2^(1)=4.839, *p*=0.028) and “Lack of training” (10 actual versus 5.9 predicted; χ^2^(1)= 5.544, *p*=0.019) as barriers to reflective practice.

Similarly, respondents who had engaged in reflective writing selected only 6 of the 14 barriers more often than expected: “Lack of time” (21 actual versus 16.5 predicted); “Concerns about confidentiality” (12 actual versus 9.8 predicted); “Negative impact on self-esteem” (2 actual versus 1.0 predicted); “Unwillingness to focus on emotions” (5 actual versus 4.4 predicted); “Fear of repercussions” (2 actual versus 1.0 predicted); and “Not supported by organizational culture” (9 actual versus 8.3 predicted). None were statistically significant. Four barriers were statistically significant, with librarians who were *not* engaged in reflective writing selecting “Lack of knowledge” (25 actual versus 17.3 predicted; χ^2^(1)=10.218, *p*=0.001); “Lack of guidance” (22 actual versus 15.3 predicted; χ^2^(1)=8.393, *p*=0.004); “Lack of training” (27 actual versus 20.4 predicted; χ^2^(1)=7.047, *p*=0.008); and “Lack of experience” (15 actual versus 10.7 predicted; χ^2^(1)=4.397, *p*=0.036) as barriers more often than expected.

One respondent provided more detail about the emotion-related barriers and the importance of preparation for reflective practice: “For me it takes a lot of detachment and self-care preparation in order to not over-identify with any feedback from participants.” Another respondent captured the desire for organizational support as evidenced by the time available for reflective practice at work: “I think it would be very important to have RP [reflective practice] supported by the organization where one is employed. I do my RP (or what passes for it) on my own time. At work I am so busy I am lucky to get time to go to the bathroom now and then.”

### Are there differences between respondents who have received formal training in reflective practice versus those who have not had training, in terms of perceived benefits or perceived barriers?

Respondents who participated in training for either reflective practice and/or reflective writing were considered to have received formal training, excluding independent or self-directed learning. Of the 106 respondents, only 7 (6.6%) reported receiving formal training in reflective practice and 8 (7.5%) reported formal training in reflective writing.

With the small number of respondents having received formal training in either reflective practice or reflective writing, it would be difficult to make statements about the relationship between training and benefits. Respondents with training selected benefits of reflective practice at least as often as expected for 22 of the 28 benefits. Benefits with the greatest difference in terms of actual numbers versus those expected as a result of the chi-square analysis were: “Catalyst for change” (6 actual versus 3.5 predicted); “Appreciation of achievements” (6 actual versus 3.9 predicted); and “Linking theory and practice” (5 actual versus 3.1 predicted). For most benefits, regardless of whether the benefit was selected more frequently by those with formal training or those without such training, the difference between actual and predicted values was less than 1.

In terms of benefits of reflective writing, those with training selected 24 of 28 benefits at least as often as expected. The 3 benefits with the greatest difference between actual values and predicted values were “Catalyst for change” (6 actual versus 3.1 predicted); “Appreciation of achievements” (6 actual versus 3.6 predicted); and “Continuing professional development” (5 actual versus 2.7 predicted). None of the differences were statistically significant.

In terms of barriers to reflective practice, respondents with formal training selected only 5 of the 14 barriers more often than predicted: “Lack of time,” “Concerns about confidentiality,” “Unwillingness to focus on emotions,” “Unwillingness to admit mistakes,” and “Fear of repercussions.” For these barriers, the differences between actual numbers versus predicted numbers were 0.5 or less. The top 3 barriers that were selected more often than expected by those *without* formal training were “Lack of training” (44 actual versus 41.6 predicted); “Not supported by organizational culture” (36 actual versus 34.2 predicted); and “Lack of motivation” (18 actual versus 16.6 predicted).

For reflective writing, only 5 of the barriers were selected more often than expected by those with training: “Lack of time,” “Concerns about confidentiality,” “No perceived benefits of reflection,” “Negative impact on self-esteem,” and “Unwillingness to focus on emotions.” For these barriers, the differences between actual numbers versus predicted numbers were less than 1.5. The top 3 barriers that were selected more often than expected by those *without* formal training were “Lack of training” (40 actual versus 36.6 predicted); “Lack of guidance” (30 actual versus 27.5 predicted); and “Lack of knowledge” (33 actual versus 31.1 predicted). The 3 barriers that were selected more often than predicted by those with training that were common to both reflective practice and writing were “Concerns about confidentiality,” “Unwilling to focus on emotions,” and “Lack of time.” For all barriers for either reflective practice or writing, none of the differences between those with training and those without training were statistically significant.

## DISCUSSION

Compared with the 2014 Greenall and Sen study, the current study found a smaller proportion of respondents identifying as reflective practitioners (77% versus 92%), which may be due to the requirement for reflective writing for all three levels of registration for the UK-based Chartered Institute of Library and Information Professionals (CILIP), whereas for health sciences librarians in the United States, there is no requirement for reflective practice in professional credentialing beyond Provisional Membership of MLA’s Academy of Health Information Professionals program.

Use of reflective writing was also slightly lower in the current study (49% versus 52%), though results were consistent in that both studies had fewer respondents who participated in reflective writing than reflective practice. A higher proportion of respondents in the current study reported reflecting on paper only (37% versus 12%) and fewer reported reflecting electronically only (21% versus 44%). In both studies, reflecting both on paper and electronically was most popular (42% versus 45%). In terms of training, respondents in the current study reported formal training far less frequently than in the Greenall and Sen study. For reflective practice, 7% versus 39% reported formal training, and for reflective writing, 8% versus 29% did so. CILIP registration may contribute to this difference because of the availability of registration-related training sessions. The top 5 benefits and barriers related to reflective practice and writing for each study are provided in Tables 3 and 4.

**Table 3:** Most commonly selected benefits of reflective practice and writing

Greenall and Sen (2014)		Miller (2019)	
**Reflective practice**			

Learning from significant incidents	(88%)	Learning from significant incidents	(85%)
Continuing professional development	(85%)	Identification of personal strengths and weaknesses	(81%)
Identification of gaps in skills and knowledge	(81%)	Identification of gaps in skills and knowledge	(75%)
Identification of personal strengths and weaknesses	(79%)	Achieving perspective	(74%)
Learning from training or educational opportunities	(77%)	Improving planning of future actions	(71%)

**Reflective writing**			

Continuing professional development	(73%)	Identification of personal strengths and weaknesses	(66%)
Learning from significant incidents	(72%)	Learning from significant incidents	(65%)
Identification of gaps in skills and knowledge	(66%)	Achieving perspective	(61%)
Improving planning of future actions	(64%)	Increasing understanding	(59%)
Increasing understanding	(63%)	Achieving clarity	(57%)

**Table 4:** Most commonly selected barriers to reflective practice and writing

Greenall and Sen (2014)		Miller (2019)	
**Reflective practice**			

Lack of time	(89%)	Lack of time	(69%)
Lack of motivation	(46%)	Lack of training	(42%)
Not supported by organizational culture	(40%)	Lack of guidance	(37%)
Lack of guidance	(35%)	Lack of knowledge	(36%)
Lack of experience	(25%)	Not supported by organizational culture	(35%)

**Reflective writing**			

Lack of time	(92%)	Lack of training	(37%)
Lack of motivation	(48%)	Lack of time	(32%)
Not supported by organizational culture	(32%)	Lack of knowledge	(31%)
Lack of training	(30%)	Lack of guidance	(28%)
Lack of guidance	(29%)	Lack of motivation	(26%)

Results of the current study suggested that exposure to reflective practice or writing might increase perceptions of benefit, while reducing perceived barriers. Exposure to reflective practice and/or writing tended to increase the likelihood that a respondent would select more benefits more often than expected and fewer barriers more often than expected. There were eleven benefits for which the differences between engaged and non-engaged respondents were statistically significant. The only statistically significant barriers were four that non-engaged respondents selected more often than expected.

Due to the small number of respondents indicating that they had participated in formal training, results of this study did not suggest that formal training was correlated with identifying a greater number of benefits of and fewer barriers to reflective practice or writing. However, in general, commonly identified barriers regardless of engagement or training—such as “Lack of knowledge,” “Lack of training,” and “Lack of guidance”—suggested that there might be a need for educational opportunities and support for health sciences librarians in reflective practice and writing.

Respondents’ comments in the current study highlighted a number of issues in reflective practice or writing that were not captured in the closed-ended questions. For example, respondents had differing opinions about whether reflective practice and/or writing should be a required part of one’s position: “Needs to be done in a supportive environment in which people feel safe and don’t feel judged. Best if not done in a required situation (such as yearly evaluation) if it is going to be turned in as a requirement” and “I think reflective practice needs peer-review/peer feedback. We do this annually, but I think it should be routine, and it should be an element in a librarian’s annual performance review.” These comments were not mutually exclusive, and they highlighted important issues to be considered by institutions considering incorporating reflective practice or writing (or the final products of those processes) as a recommended or required activity.

Comments also indicated the porous nature of the border between reflection in work settings and reflection in personal settings. Some respondents learned reflective practice or writing in personal workshops or therapy and now also used these skills in work settings. Another commented, “I am so fascinated that someone is doing a research project on this. I started keeping a journal for a yoga workshop and this practice has worked its way into my work life...but not in any formal way.”

### Limitations and future research

One of this study’s limitations is the small sample size, with a large proportion of respondents reporting that they consciously reflect on their professional practice. The high percentage (88.7%) of respondents reporting that they engage in reflective practice or writing may be due to non-response bias, with respondents choosing to complete the survey because they already had interest in or experience with these techniques to improve professional practice. Another limitation was the accidental omission of “Lack of time” as a barrier to reflective writing. This omission likely resulted in an underestimate of the extent to which respondents felt this was a barrier to reflective writing. In addition, there is a limited amount of information that can be gained with a questionnaire. The current study, together with the Greenall and Sen study, does not provide the rich detail that would increase understanding of the role of reflection in the practice of health sciences librarianship.

This study is a snapshot of the use of reflective practice and writing in a small subset of health sciences librarians. Future research should explore in more detail how and why health sciences librarians use reflective practice and writing, addressing issues such as the types of situations in which reflection is most beneficial and what specific benefits are gained, as well as the types of barriers that are most challenging and how librarians overcome them. Related to benefits and barriers, it would be important to explore how reflection can be fostered among health sciences librarians, including the role of the professional environment (credentialing, institutional requirements, etc.). It is also important to better understand the role of formal training in developing librarians’ reflective skills for the profession to design appropriate interventions and support systems. Exploration of any of these issues would provide additional insight into the use of reflective practice and writing by health sciences librarians.

### Recommendations

The current study suggests that there are opportunities to help interested health sciences librarians learn how to use reflection to improve their professional practice. While library schools are one place for this training to be offered, professional organizations can also play a role. One direct way would be to offer CE to learn reflective practice and writing, as suggested by comments such as “developing a set of exercises or curriculum aimed at the library world would be useful as a way to bring the practice into a library.”

An additional step that professional organizations can take to foster reflective practice in their programs would be to incorporate it into other CE opportunities by making questions available for pre-CE and post-CE reflection. It might be possible for MLA to provide questions to encourage deeper reflection as part of membership in the Academy of Health Information Professionals at all levels. One small example was the spring 2019 revision to the *MLA Competencies for Lifelong Learning and Professional Success*, when pre- and post-assessment questions for reflection were added to the instructions [28].

Comments from this study suggest that there is a need to support librarians in small or solo libraries in reflective practice and conversation, which mirrors comments on the Greenall and Sen study. Could a professional association provide training, facilitators, and technology to support small groups of librarians for reflective practice without geographic limitations? This support would also benefit librarians in larger libraries for whom confidentiality might be an issue.

Reflective practice or writing is an important component in the library and information science profession. It facilitates practice improvement on its own or as a part of evidence-based library and information practice. It can guide professional development and increase and enrich learning from CE, journal clubs, and other learning activities. It can support self-care by helping to reduce stress, which can lead to burnout, and improve morale and commitment to work. Most importantly, it is a tool that can be used by any librarian in any area of librarianship, regardless of length of time in the profession, to improve the service that librarians provide to patrons.

## SUPPLEMENTAL FILES

Appendix A Reflective practice and health sciences librarians questionnaire

Appendix B Ranked benefits and barriers by work environment based on counts of survey responses

## 

**Jolene M. Miller, MLS, AHIP**, jolene.miller@utoledo.edu, https://orcid.org/0000-0003-4422-2708, Director, Mulford Health Science Library, and Assistant Professor, Library Administration, University of Toledo, Toledo, OH
