# Supplementary material for: Reflective practice and health sciences librarians: engagement, benefits, and barriers
Source: J Med Libr Assoc. 2020 Jan 1;108(1):17–28. doi: 10.5195/jmla.2020.777 (PMC6919986; doi:10.5195/jmla.2020.777)
Supplement: Appendix A [file jmla-108-17-s001.pdf]

JMLA Journal of the Medical Library Association

SC

Supplemental content to
J Med Libr Assoc. Jan;108(1):dx.doi.org/10.5195/jmla.2020.777
www.jmla.mlanet.org
© Miller 2020

Reflective practice and health sciences librarians: engagement, benefits, and barriers

Jolene M. Miller, MLS, AHIP

APPENDIX A

Reflective practice and health sciences librarians questionnaire

Adult Research: Informed consent information

Study: "Utilization of intentional reflective practice among health sciences librarians"

Principal Investigator: Jolene M. Miller, MLS, AHIP, director, Mulford Health Science Library; 419.383.4959

Purpose: You are invited to participate in the research project, titled "Utilization of Intentional Reflective Practice among Health Sciences Librarians," which is being conducted at the University of Toledo by Jolene Miller. The purpose of this study is to better understand how medical/health sciences librarians are using reflective practice and reflective writing to become better librarians.

Description of Procedures: This research will take place online, using a modified version of the 2014 Greenall and Sen questionnaire. The survey asks about perceived benefits of and barriers to reflective practice and writing and how you may have learned about reflective practice and writing. Your participation will take less than 20 minutes to complete the survey. It will remain open until April 20, 2017.

Potential Risks: There are minimal risks to participation in this study. The survey is anonymous, and it does not ask for any identifying information. No one will know that you completed the survey or what you answered. You do not have to answer all of the questions, and you can quit the survey at any time.

Potential Benefits: The only direct benefit to you if you participate in this research may be that you learn more about the use of intentional reflective practice and reflective writing in becoming a better librarian. Others may benefit from the results of this research.

Confidentiality: The researcher will make every effort to prevent anyone else from knowing that you provided this information or what that information is. To achieve this, the online survey is anonymous. Even the researcher will not know that you participated in the study.

Voluntary Participation: Your refusal to participate in this study will involve no penalty or loss of benefits to which you are otherwise entitled and will not affect your relationship with the University of Toledo. In addition, you may discontinue participation at any time without any penalty or loss of benefits.

Contact Information: Before you decide to accept this invitation to take part in this study, you may ask any questions that you might have. If you have any questions at any time before, during, or after your participation, you should contact Jolene Miller, 419.383.4959. If you have questions beyond those she answered or about your rights as a research subject or research-related injuries, please feel free to contact the Social, Behavioral, and Educational Institutional Review Board chair at 419.530.2844.

Reflective practice is an activity undertaken by professionals to enable them to deal with complex situations by evaluating actual or possible events or scenarios to gain insight and learn from experience. This could involve thinking alone about some aspect of practice, group discussion with colleagues,

Supplemental content to
J Med Libr Assoc. Jan;108(1):dx.doi.org/10.5195/jmla.2020.777
www.jmla.mlanet.org
© Miller 2020

dialogue with another person, or writing/journaling. Note: If you have any comments or clarifications about any of the survey questions, you can enter them in the last question of the survey.

1. Do you consider yourself to be a reflective practitioner?
- o Yes
- o Sometimes
- o No

2. Do you consciously spend time reflecting on your professional practice?
- o Yes
- o Sometimes
- o No

3. Do you consciously determine how the outcome of this reflection will affect your current or future practice?
- o Yes
- o Sometimes
- o No

These questions ask about reflective practice that involves thinking alone, sharing in group discussion with colleagues, or talking to one other person. For this section, please do NOT consider reflective writing. There is a separate section on reflective writing.

4. Have you ever had any training in professional or personal reflective practice?
- o Yes
- o No

5. Where did you learn about reflective practice? Select all that apply.
- o During library school
- o Continuing education program sponsored by a professional organization
- o Continuing education program sponsored by my institution
- o Mentoring relationship
- o Educational program for personal development
- o Self-directed, independent learning
- o Other; please specify: ____________________

6. Whether or not you consider yourself to be a reflective practitioner, which of the following do you think are benefits of engaging in reflective practice? Select all that apply.
- o Continuing professional development
- o Learning from significant incidents
- o Learning from training or educational opportunities
- o Identification of gaps in skills and knowledge
- o Identification of personal strengths and weaknesses
- o Identification of goals
- o Increasing knowledge
- o Increasing understanding
- o Linking theory and practice
- o Improving planning of future actions
- o Improving professional judgments
- o Improving critical thinking

SC
Supplemental content to
J Med Libr Assoc. Jan;108(1):dx.doi.org/10.5195/jmla.2020.777
www.jmla.mlanet.org
© Miller 2020

- Solving dilemmas
- Achieving perspective
- Achieving clarity
- Understanding and expressing emotions
- Managing stress
- Understanding the perspective of others
- Improving working relationships
- Improving professional practice
- Identify the need to change
- Catalyst for change
- Personal empowerment
- Emancipation
- Self-development
- Appreciation of achievements
- Sharing experiences with others
- Demonstrating professional practice to others
- Other; please specify: ____________________

7. Which of the following do you think are barriers that prevent you from engaging in reflective practice to the extent that you prefer? Select all that apply.

- Lack of time
- Lack of knowledge
- Lack of guidance
- Lack of training
- Lack of skill
- Lack of experience
- Lack of motivation
- Concerns about confidentiality
- No perceived benefits of reflection
- Negative impact on self-esteem
- Unwillingness to focus on emotions
- Unwillingness to admit mistakes
- Fear of repercussions
- Not supported by organizational culture
- Other; please specify: ____________________

8. What else would you like to share about reflective practice?

Reflective writing is a way of recording reflections, which can be paper or electronic, and can be completed regularly or irregularly. It should involve analysis and evaluation of events from different perspectives and include plans for future actions resulting from the reflection.

9. Do you engage in reflective writing?

- Yes
- Sometimes
- No

JMLA Journal of the Medical Library Association

SC
Supplemental content to
J Med Libr Assoc. Jan;108(1):dx.doi.org/10.5195/jmla.2020.777
www.jmla.mlanet.org
© Miller 2020

10. What form does this reflective writing take?
- o Paper
- o Electronic
- o Both paper and electronic

11. In general, how widely do you share your reflective writing?
- o I don't share it; it is private.
- o I share it widely; it is public (such as on a blog).
- o I share selective reflections widely.
- o I share it with selected people (such as a mentor).
- o Other; please specify: ____________________

12. Why do you undertake reflective writing? Select all that apply.
- o To fulfill the requirements of position/institution
- o To fulfill the requirements of a course of study
- o To fulfill the requirements of a professional qualification/credential
- o To support my growth as a professional
- o Other; please specify: ____________________

13. Have you ever had any training in professional or personal reflective writing?
- o Yes
- o No

14. Where did you learn about reflective writing? Select all that apply.
- o During library school
- o Continuing education program sponsored by a professional organization
- o Continuing education program sponsored by my institution
- o Mentoring relationship
- o Educational program for personal development
- o Self-directed, independent learning
- o Other; please specify: ____________________

15. Whether or not you consider yourself to be a reflective practitioner, which of the following do you think are benefits of engaging in reflective writing? Select all that apply.
- o Continuing professional development
- o Learning from significant incidents
- o Learning from training or educational opportunities
- o Identification of gaps in skills and knowledge
- o Identification of personal strengths and weaknesses
- o Identification of goals
- o Increasing knowledge
- o Increasing understanding
- o Linking theory and practice
- o Improving planning of future actions
- o Improving professional judgments
- o Improving critical thinking
- o Solving dilemmas
- o Achieving perspective
- o Achieving clarity
- o Understanding and expressing emotions
- o Managing stress

SC
Supplemental content to
J Med Libr Assoc. Jan;108(1):dx.doi.org/10.5195/jmla.2020.777
www.jmla.mlanet.org
© Miller 2020

- Understanding the perspective of others
- Improving working relationships
- Improving professional practice
- Identify the need to change
- Catalyst for change
- Personal empowerment
- Emancipation
- Self-development
- Appreciation of achievements
- Sharing experiences with others
- Demonstrating professional practice to others
- Other; please specify: ____________________

16. Which of the following do you think are barriers that prevent you from engaging in reflective writing to the extent that you prefer? Select all that apply.

- Lack of knowledge
- Lack of guidance
- Lack of training
- Lack of skill
- Lack of experience
- Lack of motivation
- Concerns about confidentiality
- No perceived benefits of reflection
- Negative impact on self-esteem
- Unwillingness to focus on emotions
- Unwillingness to admit mistakes
- Fear of repercussions
- Not supported by organizational culture
- Other; please specify: ____________________

17. What else would you like to share about reflective writing?

____

18. In which type of library/information science environment are you currently employed?

- Hospital/medical center
- Academic health sciences/academic medical center
- Nursing/allied health school
- College/university
- Medical or health sciences association, society, nonprofit
- Government, including VA and military units
- Self-employed/consultant
- School of library or information studies
- Research or health research center
- Not currently employed (student, retired, etc.)
- Other; please specify: ____________________

SC
Supplemental content to
J Med Libr Assoc. Jan;108(1):dx.doi.org/10.5195/jmla.2020.777
www.jmla.mlanet.org
© Miller 2020

19. How long have you been employed in library/information environments?
- o Not yet or not at all
- o Some, but less than 5 years
- o 5 to 10 years
- o 11 to 15 years
- o 16 to 20 years
- o 21 to 25 years
- o Over 25 years

20. What else do you think the researcher should know about reflective practice and writing in medical/health sciences librarianship? Do you have any comments or clarifications regarding any previous question(s)?
