# Supplementary material for: Reflective practice and health sciences librarians: engagement, benefits, and barriers
Source: J Med Libr Assoc. 2020 Jan 1;108(1):17–28. doi: 10.5195/jmla.2020.777 (PMC6919986; doi:10.5195/jmla.2020.777)
Supplement: Appendix B [file jmla-108-17-s002.pdf]

JMLA Journal of the Medical Library Association

SC
Supplemental content to
J Med Libr Assoc. Jan;108(1):dx.doi.org/10.5195/jmla.2020.777
www.jmla.mlanet.org
© Miller 2020

Reflective practice and health sciences librarians: engagement, benefits, and barriers

Jolene M. Miller, MLS, AHIP

APPENDIX B
Ranked benefits and barriers by work environment based on counts of survey responses

Benefits: Combined reflective practice and reflective writing (n=212)

Totals for all work environments	
1. Learning from significant incidents	159
2. Identification of personal strengths and weaknesses	156
3. Achieving perspective	143
4. Increasing understanding	137
5. Identification of gaps in skills and knowledge	135
Hospital/medical center library	
1. Learning from significant incidents	51
2. Achieving perspective	44
3. Identification of personal strengths and weaknesses	42
3. Increasing understanding	42
3. Self-development	42
Academic health center/academic medical center library	
1. Identification of personal strengths and weaknesses	72
2. Learning from significant incidents	69
3. Identification of gaps in skills and knowledge	63
4. Achieving perspective	61
5. Self-development	59
5. Improving planning of future actions	59
College/university library	
1. Identification of personal strengths and weaknesses	24
2. Identification of gaps in skills and knowledge	23
3. Achieving clarity	22
4. Achieving perspective	21
4. Improving planning of future actions	21
Other types of libraries	
1. Identification of gaps in skills and knowledge	20
2. Learning from significant incidents	19
3. Identification of personal strengths and weaknesses	18
3. Identification of goals	18
4. Achieving perspective	17
4. Increasing understanding	17

JMLA Journal of the Medical Library Association

Supplemental content to
J Med Libr Assoc. Jan;108(1):dx.doi.org/10.5195/jmla.2020.777
www.jmla.mlanet.org
© Miller 2020

Benefits: Reflective practice (n=106)

<u>Totals for all work environments</u>	
1. Learning from significant incidents	90
2. Identification of personal strengths and weaknesses	86
3. Identification of gaps in skills and knowledge	79
4. Achieving perspective	78
5. Improving planning of future actions	75
<u>Hospital/medical center library</u>	
1. Learning from significant incidents	28
2. Achieving perspective	26
3. Identification of personal strengths and weaknesses	24
4. Increasing understanding	23
4. Solving dilemmas	23
<u>Academic health center/academic medical center library</u>	
1. Identification of personal strengths and weaknesses	40
2. Learning from significant incidents	39
3. Identification of gaps in skills and knowledge	38
4. Improving professional practice	36
5. Improving planning of future actions	34
5. Self-development	34
<u>College/university library</u>	
1. Identification of personal strengths and weaknesses	12
1. Learning from significant incidents	12
1. Identification of gaps in skills and knowledge	12
2. Improving planning of future actions	11
2. Achieving perspective	11
2. Achieving clarity	11
2. Improving professional judgments	11
2. Understanding perspective of others	11
<u>Other types of libraries</u>	
1. Learning from significant incidents	11
1. Identification of gaps in skills and knowledge	11
2. Identification of personal strengths and weaknesses	10
2. Identification of goals	10
3. Achieving perspective	9
3. Increasing understanding	9
3. Improving professional practice	9
3. Solving dilemmas	9
3. Sharing experiences with others	9

JMLA Journal of the Medical Library Association

Supplemental content to
J Med Libr Assoc. Jan;108(1):dx.doi.org/10.5195/jmla.2020.777
www.jmla.mlanet.org
© Miller 2020

Benefits: Reflective writing (n=106)

Totals for all work environments	
1. Identification of personal strengths and weaknesses	70
2. Learning from significant incidents	69
3. Achieving perspective	65
4. Increasing understanding	63
5. Achieving clarity	60
Hospital/medical center library	
1. Learning from significant incidents	23
2. Managing stress	20
2. Self-development	20
3. Increasing understanding	19
4. Identification of personal strengths and weaknesses	18
4. Achieving perspective	18
4. Solving dilemmas	18
Academic health center/academic medical center library	
1. Identification of personal strengths and weaknesses	32
2. Learning from significant incidents	30
3. Achieving perspective	29
4. Managing stress	27
5. Increasing understanding	26
5. Achieving clarity	26
College/university library	
1. Identification of personal strengths and weaknesses	12
2. Achieving clarity	11
2. Identification of gaps in skills and knowledge	11
3. Achieving perspective	10
3. Increasing understanding	10
3. Improving planning of future actions	10
Other types of libraries	
1. Identification of gaps in skills and knowledge	9
2. Identification of personal strengths and weaknesses	8
2. Achieving perspective	8
2. Increasing understanding	8
2. Identification of goals	8
2. Learning from significant incidents	8

JMLA Journal of the Medical Library Association

Supplemental content to
J Med Libr Assoc. Jan;108(1):dx.doi.org/10.5195/jmla.2020.777
www.jmla.mlanet.org
© Miller 2020

Barriers: Combined reflective practice and reflective writing (n=212)

<u>Totals for all work environments</u>	
1. Lack of time	107
2. Lack of training	83
3. Lack of knowledge	71
4. Lack of guidance	69
5. Not supported by organizational culture	54
<u>Hospital/medical center library</u>	
1. Lack of training	34
2. Lack of time	29
3. Lack of knowledge	27
3. Lack of guidance	27
4. Lack of motivation	18
<u>Academic health center/academic medical center library</u>	
1. Lack of time	52
2. Lack of training	31
3. Lack of knowledge	30
4. Not supported by organizational culture	28
5. Lack of guidance	25
<u>College/university library</u>	
1. Lack of time	16
2. Lack of training	10
3. Lack of guidance	8
4. Lack of knowledge	7
5. Not supported by organizational culture	6
5. Lack of motivation	6
<u>Other types of libraries</u>	
1. Lack of time	10
2. Lack of guidance	9
3. Lack of training	8
4. Lack of knowledge	7
4. Concerns about confidentiality	7
4. Unwillingness to focus on emotions	7

SC
Supplemental content to
J Med Libr Assoc. Jan;108(1):dx.doi.org/10.5195/jmla.2020.777
www.jmla.mlanet.org
© Miller 2020

Barriers: Reflective practice (n=106)

Totals for all work environments	
1. Lack of time	73
2. Lack of training	44
3. Lack of guidance	39
4. Lack of knowledge	38
5. Not supported by organizational culture	37
Hospital/medical center library	
1. Lack of time	21
2. Lack of training	19
3. Lack of guidance	14
3. Lack of knowledge	14
4. Lack of skill	10
Academic health center/academic medical center library	
1. Lack of time	33
2. Not supported by organizational culture	21
3. Lack of training	17
3. Lack of knowledge	17
4. Lack of guidance	15
College/university library	
1. Lack of time	12
2. Not supported by organizational culture	4
2. Lack of training	4
2. Lack of guidance	4
3. Lack of knowledge	3
3. Lack of motivation	3
Other types of libraries	
1. Lack of time	7
2. Lack of guidance	6
3. Lack of training	4
3. Lack of knowledge	4
4. Not supported by organizational culture	3
4. Concerns about confidentiality	3
4. Unwillingness to focus on emotions	3

Supplemental content to
J Med Libr Assoc. Jan;108(1):dx.doi.org/10.5195/jmla.2020.777
www.jmla.mlanet.org
© Miller 2020

Barriers: Reflective writing (n=106)
Note: "Lack of time" was accidentally omitted from the list of barriers to reflective writing. Data were gathered from comments that mentioned "Lack of time."

Totals for all work environments	
1. Lack of training	39
2. Lack of time	34
3. Lack of knowledge	33
4. Lack of guidance	30
5. Lack of motivation	27

Hospital/medical center library	
1. Lack of training	15
2. Lack of knowledge	13
2. Lack of guidance	13
3. Lack of motivation	11
4. Lack of time	8

Academic health center/academic medical center library	
1. Lack of time	19
2. Lack of training	14
3. Lack of knowledge	13
3. Lack of experience	13
4. Lack of skill	12

College/university library	
1. Lack of training	6
2. Lack of time	4
2. Lack of knowledge	4
2. Lack of skill	4
2. Lack of guidance	4

Other types of libraries	
1. Lack of training	4
1. Unwillingness to focus on emotions	4
1. Concerns about confidentiality	4
2. Lack of time	3
2. Lack of knowledge	3
2. Lack of guidance	3
2. Lack of motivation	3
2. Not supported by organizational culture	3
2. Lack of experience	3
2. Unwillingness to admit mistakes	3
